# Association of ideal cardiovascular health at age 50 with incidence of dementia: 25 year follow-up of Whitehall II cohort study

**DOI:** 10.1136/bmj.l4414

**Published:** 2019-07-31

**Authors:** Séverine Sabia, Aurore Fayosse, Julien Dumurgier, Alexis Schnitzler, Jean-Philippe Empana, Klaus P Ebmeier, Aline Dugravot, Mika Kivimäki, Archana Singh-Manoux

**Affiliations:** 1 Inserm U1153, Epidemiology of Ageing and Neurodegenerative diseases, Université de Paris, 75010 Paris, France; 2 Department of Epidemiology and Public Health, University College London, London, UK; 3 Cognitive Neurology Center, Lariboisière – Fernand Widal Hospital, AP-HP, Université Paris Diderot, Sorbonne Paris Cité, Paris, France; 4 Inserm, U970, Integrative Epidemiology of Cardiovascular Disease, Paris Descartes University, Paris, France; 5 Department of Psychiatry, University of Oxford, Oxford, UK; 6 Helsinki Institute of Life Sciences, University of Helsinki, Helsinki, Finland

## Abstract

**Objectives:**

To examine the association between the Life Simple 7 cardiovascular health score at age 50 and incidence of dementia.

**Design:**

Prospective cohort study.

**Setting:**

Civil service departments in London (Whitehall II study; study inception 1985-88).

**Participants:**

7899 participants with data on the cardiovascular health score at age 50.

**Exposures:**

The cardiovascular health score included four behavioural (smoking, diet, physical activity, body mass index) and three biological (fasting glucose, blood cholesterol, blood pressure) metrics, coded on a three point scale (0, 1, 2). The cardiovascular health score was the sum of seven metrics (score range 0-14) and was categorised into poor (scores 0-6), intermediate (7-11), and optimal (12-14) cardiovascular health.

**Main outcome measure:**

Incident dementia, identified through linkage to hospital, mental health services, and mortality registers until 2017.

**Results:**

347 incident cases of dementia were recorded over a median follow-up of 24.7 years. Compared with an incidence rate of dementia of 3.2 (95% confidence interval 2.5 to 4.0) per 1000 person years among the group with poor cardiovascular health, the absolute rate differences per 1000 person years were −1.5 (95% confidence interval −2.3 to −0.7) for the group with intermediate cardiovascular health and −1.9 (−2.8 to −1.1) for the group with optimal cardiovascular health. Higher cardiovascular health score was associated with a lower risk of dementia (hazard ratio 0.89 (0.85 to 0.95) per 1 point increment in the cardiovascular health score). Similar associations with dementia were observed for the behavioural and biological subscales (hazard ratios per 1 point increment in the subscores 0.87 (0.81 to 0.93) and 0.91 (0.83 to 1.00), respectively). The association between cardiovascular health at age 50 and dementia was also seen in people who remained free of cardiovascular disease over the follow-up (hazard ratio 0.89 (0.84 to 0.95) per 1 point increment in the cardiovascular health score).

**Conclusion:**

Adherence to the Life Simple 7 ideal cardiovascular health recommendations in midlife was associated with a lower risk of dementia later in life.

## Introduction

Pathophysiological hallmarks of dementia appear 15-20 years before clinical symptoms,^1^ highlighting the need for a long follow-up to identify risk factors and protective factors. Guidelines for prevention of dementia recommend targeting midlife cardiovascular risk factors.^2^ ^3^ Much of the evidence is on individual cardiovascular risk factors considered one at a time, although the importance of their clustering is increasingly recognised. In 2010 the American Heart Association (AHA) proposed the ideal cardiovascular health score, also referred to as Life’s Simple 7, composed of four behavioural and three biological metrics for primordial prevention of cardiovascular disease.^4^ People with higher cardiovascular health scores have a lower risk of cardiometabolic diseases, such as type 2 diabetes, coronary heart disease, and stroke, than do those with lower scores.^5^ ^6^

The cardiovascular health score has recently been put forward as a tool for the promotion of brain health.^7^ In studies on older populations, a higher cardiovascular health score has been shown to be associated with slower cognitive decline and reduced risk of dementia,^8^ although the evidence is inconsistent across studies.^9^ ^10^ ^11^ An important source of uncertainty in the evidence is failure to consider the long preclinical period preceding the diagnosis of dementia.^1^ In studies with a short follow-up, risk factor levels might be affected by the preclinical phase of dementia, as shown for obesity and hypertension,^12^ ^13^ which are two of the seven metrics included in the cardiovascular health score.

Using data from the Whitehall II cohort study spanning nearly three decades, we aimed to investigate the association of cardiovascular health score assessed at age 50 with incidence of late life dementia; to assess whether cardiovascular health score at age 50 is associated with incidence of dementia in people who remain free of cardiovascular disease until diagnosis of dementia; and, in a subcohort of the Whitehall study, examine the association of cardiovascular health score at age 50 with brain volume assessed 20 years later using magnetic resonance imaging.

## Methods

### Study population

The Whitehall II study is an ongoing cohort study of 10 308 people (6895 men and 3413 women, aged 35-55) recruited from the British civil service in 1985-88.^14^ Baseline assessment consisted of a questionnaire and a structured clinical evaluation composed of measures of anthropometric, cardiovascular, and metabolic risk factors and diseases. Subsequent follow-up clinical examinations have taken place approximately every four to five years (1991, 1997, 2002, 2007, 2012, and 2015), with each wave taking two years to complete. Written informed consent from participants was renewed at each contact.

### Measures

#### Cardiovascular health at age 50

We used data collected at the first four clinical examinations of the study (1985 (age range 35-55 years), 1991 (40-64 years), 1997 (45-69 years), and 2002 (50-74 years)) to extract information on the seven cardiovascular health metrics at age 50 for every participant, allowing a five year margin either way.

Smoking status (current, past, never), physical activity (hours of moderate and vigorous physical activity per week), and diet (fruit and vegetable consumption, types of bread) were assessed using questionnaires. A trained nurse used standard protocols to collect data on body mass index (weight/height^2^ in kg/m^2^), fasting glucose concentration, blood cholesterol concentration, and blood pressure during the clinical examination. Blood pressure was taken as the mean of two measurements using a Hawksley random zero sphygmomanometer (1985, 1991, and 1997) and OMRON HEM 907 (2002) with the participant in a sitting position after five minutes of rest. Weight was measured in underwear to the nearest 0.1 kg on Soehnle electronic scales with digital readout (Leifheit, Nassau, Germany), and height was measured in bare feet to the nearest 1 mm by using a stadiometer with the participant standing erect with the head in the Frankfurt plane. Venous blood samples were taken after at least five hours of fasting, and serum obtained after centrifugation was refrigerated at 4°C and assayed within 72 hours of the blood draw. Total cholesterol was measured using a Cobas Fara centrifugal analyzer (Roche Diagnostics, Nutley, NJ).

We categorised all metrics into three levels (coded as poor=0, intermediate=1, and optimal=2), following the AHA’s recommendations,^4^ apart from smoking and diet, for which we adapted the categorisation to reflect data available in the study (table 1). For smoking, we split ex-smokers into optimal and intermediate levels depending on whether they had stopped smoking before or after the previous wave of data collection (corresponding to a four to five year gap, instead of one year). For the diet metric, we used information on consumption of fruit and vegetables and type of bread, as this information was available from all waves of data collection.

**Table 1:** Definition of cardiovascular health metrics

Metrics	Poor level (score=0)	Intermediate level (score=1)	Optimal level (score=2)
Smoking	Current smoker	Stopped in past 5 years	Never smoked or stopped >5 years ago
Diet	Consumption of fruit and vegetable less than twice a day AND no consumption of high fibre bread	Consumption of fruit and vegetable twice a day or more OR consumption of high fibre bread	Consumption of fruit and vegetable twice a day or more AND consumption of high fibre bread
Physical activity	No moderate or vigorous physical activity	1-149 min/week of moderate activity OR 1-74 min/week of vigorous activity OR 1-149 min/week of moderate and vigorous activity	≥150 min/week of moderate activity OR ≥75 min/week of vigorous activity OR ≥150 min/week of moderate and vigorous activity
Body mass index	≥30	25-29.9	<25
Fasting glucose	≥126 mg/dL	<100 mg/dL treated OR 100-125 mg/dL	<100 mg/dL untreated
Blood cholesterol	≥240 mg/dL	<200 mg/dL treated OR 200-239 mg/dL	<200 mg/dL untreated
Systolic and diastolic blood pressure	SBP ≥140 mm Hg OR DBP ≥90 mm Hg	SBP <120 mm Hg and DBP <80 mm Hg treated OR SBP 120-139 OR DBP 80-89 mm Hg	SBP <120 mm Hg and DBP <80 mm Hg untreated

DBP=diastolic blood pressure; SBP=systolic blood pressure.

We used the sum of each metric to calculate the cardiovascular health score, ranging from 0 to 14 with higher scores corresponding to better cardiovascular health. We categorised this score as poor for scores ranging from 0 to 6 (corresponding to less than one standard deviation from the mean), intermediate for scores ranging from 7 to 11 (+/−1 SD from the mean), and optimal for scores between 12 and 14 (>1 SD from the mean). We also derived the number of cardiovascular health metrics at optimal level, ranging from zero (none) to seven (all metrics at optimal level).

We calculated a behavioural score as the sum of the behavioural metrics (smoking, physical activity, diet, body mass index) ranging from 0 (worst) to 8 (best) and a biological score as the sum of the biological metrics (glucose, cholesterol, blood pressure) ranging from 0 (worst) to 6 (best), as recommended by the AHA.^4^

#### Dementia

All residents in England, Scotland, and Wales have a unique National Health Service (NHS) identification number, which we used to link all participants to electronic health records. We used three registers (the national hospital episode statistics (HES) database, the Mental Health Services Data Set, and the mortality register) for ascertainment of dementia using ICD-10 (international classification of disease, 10th revision) codes F00-F03, F05.1, G30, and G31. Record linkage was available until 31 March 2017. The NHS provides most of the healthcare, including outpatient and inpatient care. The sensitivity of dementia in the HES data is 78.0%, and the specificity is 92.0%.^15^ In addition, we used the Mental Health Services Data Set, a national database containing information on dementia for people in contact with mental health services in hospitals, outpatient clinics, and the community. Cause specific mortality data came from the British national mortality register (National Health Services Central Registry). Date of dementia was set at the first record of diagnosis of dementia in any of the three databases used for ascertainment.

#### Cardiovascular disease

Coronary heart disease was identified by study specific assessments (12 lead resting electrocardiogram recording, coded using the Minnesota system), self reported coronary heart disease (verified in medical records), and linkage to HES (ICD-9 codes 410-414, ICD-10 codes I20-I25, or procedures K40–K49, K50, K75, U19). Stroke was determined using linkage to HES (ICD-9 430, 431, 434, 436; ICD-10 I60-I64).

#### Covariates

Covariates assessed at age 50 included exact age, sex, ethnicity (white, non-white), marital status (married/cohabiting, other), and socioeconomic status according to occupational position (high, intermediate, and low, representing income and status at work) and education (less than primary school (up to age 11), lower secondary school (up to age 16), higher secondary school (up to age 18), university, and higher university degree). In sensitivity analysis, we also used data on apolipoprotein E ε4.

#### Brain volume

Between 2012 and 2016, a magnetic resonance imaging substudy was implemented in 771 participants of the Whitehall II study. Details of the imaging protocol and the analysis pipelines have been published previously.^16^ In brief, multimodal magnetic resonance imaging scans were acquired at the Oxford Centre for Functional MRI of the Brain (FMRIB Centre) by using a 3-tesla magnetic resonance imaging scanner (MAGNETOM Verio; Siemens Healthineers, Erlangen, Germany) with a 32 channel head coil. Structural images were acquired using a high resolution, three dimensional, T1 weighted sequence (repetition time 2530 ms, echo time 7.37 ms, flip angle 78 degrees, field of view 256 mm, voxel dimensions 1.0 mm isotropic). Data processing and analysis of magnetic resonance imaging was done using FMRIB Software Library (FMRIB, Oxford, UK). Brain tissues were segmented using FMRIB’s automated segmentation tool, which allows extraction of measures of total grey matter, white matter, and cerebrospinal fluid, which were summed to calculate intracranial volume. All volumes were normalised by converting them to percentages of the intracranial volume. Burden of white matter hyperintensities was estimated by using the Fazekas visual rating scale.^17^ A score (range 0-3) was assigned for deep white matter and periventricular areas depending on the size, location, and confluence of lesions. Total burden of white matter hyperintensities was estimated by using the sum of the two scores (range 0-6). Three clinicians independently defined hippocampal atrophy according to visual rating (Scheltens score,^18^ range 0-8) and reached a consensus.

### Statistical analysis

#### Association of cardiovascular health at age 50 with incidence of dementia

We examined the association of cardiovascular health score at age 50 with risk of incident dementia by using Cox regression with date of entry being the date of clinical examination at age 50. We followed participants to the date of record of dementia, death (to account for competing risk of mortality), or 31 March 2017, whichever came first. We first examined the shape of the association of the continuous cardiovascular health score with risk of dementia by using restricted cubic spline regressions with Harrell knots,^19^ Stata command xblc,^20^ with score 9 (mean cardiovascular health score) as the reference (hazard ratio=1). In addition, we examined the association with the cardiovascular health score categorised as poor (score 0-6), intermediate (score 7-11), and optimal (score 12-14) and then used cardiovascular health score as a linear variable to examine the risk reduction associated with a 1 point increment. We repeated the analysis for an increment of one additional cardiovascular health metric at the optimal level and for the behavioural and biological subscale scores. We used age as the timescale and adjusted analyses first for sex, ethnicity, educational level, occupational position, and marital status and then for time dependent cardiovascular disease. For the subsample with available data on apolipoprotein E ε4 genotype, we also adjusted for this variable.

We used inverse probability weighting to account for missing data on cardiovascular health metrics.^21^ This involved calculation of the probability of being included in the analytical sample by using data on the seven cardiovascular health metrics, sociodemographic factors, chronic conditions (coronary heart disease, stroke, diabetes, chronic obstructive pulmonary disease, cancer), and dementia status over the follow-up, including the interactions between dementia status and the cardiovascular health metrics. We used the inverse of these probabilities to weight the results from the Cox regression.

#### Role of cardiovascular health score at age 50 in transitions between healthy state, cardiovascular disease, and dementia

In subsequent analyses, we used multi-state models to examine the role of cardiovascular disease (incident coronary heart disease or stroke) over the follow-up in the association between cardiovascular health score at age 50 and risk of dementia. These models allow simultaneous estimation of the association of cardiovascular health score at age 50 with risk of cardiovascular disease over the follow-up (transition A), the association of cardiovascular health score at age 50 with risk of dementia in people without cardiovascular disease over the follow-up (transition B), and the association of cardiovascular health score at age 50 with risk of dementia following diagnosis of cardiovascular disease (transition C). As in the previous analyses, models were adjusted for sociodemographic factors and weighted using inverse probability weighting.

#### Association between cardiovascular health score and brain volume

In the magnetic resonance imaging analysis, we examined the association of cardiovascular health at age 50 with whole brain volume (sum of grey and white matter), grey matter volume, and white matter volume, as well as with levels of white matter hyperintensities and hippocampal atrophy on average 20 years later by using linear regression adjusted for age at magnetic resonance imaging assessment, the magnetic resonance imaging scanner, and sociodemographic factors at age 50.

We ran the multi-state models by using the “mstate” package of R software. For all other analyses, we used Stata 15.1. We rejected the null hypothesis for two sided values of P<0.05.

### Patient and public involvement

Participants of the Whitehall II study were not involved in setting the research question or the outcome measures, nor were they involved in developing plans for recruitment, design, or implementation of the study. No participants were asked for advice on interpretation or writing up of results. However, all results are disseminated to study participants via newsletters and our website, which has a participant portal (https://www.ucl.ac.uk/epidemiology-health-care/research/epidemiology-and-public-health/research/whitehall-ii/participants-area). The dissemination plan targets a large audience, including members of the public, patients, and health professionals, and will be achieved through various channels, including media outreach via press release from the Inserm and University College London, networks, and social media.

## Results

**Fig 1:**
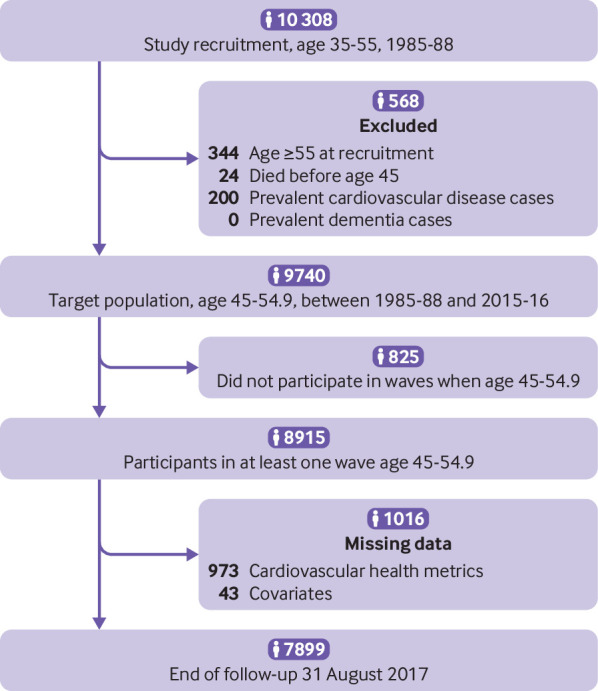
Flowchart of sample selection

Among the 9740 participants free of cardiovascular disease and dementia at age 50 (target population), data on all cardiovascular health metrics at age 50 (mean age at assessment 50.4 (SD 2.3) years) were available for 7899 participants (fig 1). Compared with those not included in the analysis (n=1841), study participants were older at baseline (45.3 *v* 41.6 years) and were more likely to be men (67.6% (n=5337) *v* 64.2% (n=1182)) and to develop dementia over the follow-up period (4.4% (n=347) *v* 3.3% (n=61)) (all P<0.05), leading us to use inverse probability weighting to take these differences into account in the analyses.

Among the 7899 study participants, a total of 347 cases of dementia were recorded over a median follow-up of 24.7 (interquartile range 19.1-29.3) years. Mean age at diagnosis of dementia was 75.3 (SD 5.3; interquartile range 72.3-79.4) years. Table 2 shows characteristics of the study sample at age 50. The proportion of women, non-white participants, and participants in the lower socioeconomic group was higher in the group of dementia cases compared with participants who did not develop dementia during follow-up. Participants with higher cardiovascular health score, denoting better health, were more likely to be male, white, married/cohabiting, and from the higher educational and occupational groups. Further distribution of the cardiovascular health metrics and by categories of the cardiovascular health score are shown in supplementary table A.

**Table 2:** Sample characteristics at age 50 (n=7899). Values are numbers (percentages) unless stated otherwise

Characteristics	Cardiovascular health score at age 50			P value	Dementia status at end of follow-up		P value
	Poor (0-6; n=978)	Intermediate (7-11; n=5997)	Optimal (12-14; n=924)		No dementia (n=7552)	Dementia (n=347)	
Sex:				<0.001			<0.001
Men	578 (59.1)	4119 (68.7)	640 (69.3)		5143 (68.1)	194 (55.9)	
Women	400 (40.9)	1878 (31.3)	284 (30.7)		2409 (31.9)	153 (44.1)	
Ethnicity:				<0.001			<0.001
White	823 (84.1)	5408 (90.2)	884 (95.7)		6823 (90.3)	292 (84.1)	
Non-white	155 (15.8)	589 (9.8)	40 (4.3)		729 (9.7)	55 (15.9)	
Education:				<0.001			<0.001
Less than primary school	137 (14.0)	633 (10.6)	67 (7.3)		776 (10.3)	61 (17.6)	
Lower secondary school	459 (46.9)	2185 (36.4)	257 (27.8)		2751 (36.4)	150 (43.2)	
Higher secondary school	227 (23.2)	1586 (26.4)	232 (25.1)		1977 (26.2)	68 (19.6)	
University	116 (11.9)	1226 (20.4)	266 (28.8)		1560 (20.7)	48 (13.8)	
Higher university degree	39 (4.0)	367 (6.1)	102 (11.0)		488 (6.5)	20 (5.8)	
Occupational position:				<0.001			<0.001
Low	343 (35.1)	1091 (18.2)	78 (8.4)		1378 (18.2)	134 (38.6)	
Intermediate	417 (42.6)	2620 (43.7)	372 (40.3)		3283 (43.5)	126 (36.3)	
High	218 (22.3)	2286 (38.1)	474 (51.3)		2891 (38.3)	87 (25.1)	
Married/cohabiting:				<0.001			0.10
No	295 (30.2)	1430 (23.8)	214 (23.2)		1841 (24.4)	98 (28.2)	
Yes	683 (69.8)	4567 (76.2)	710 (76.8)		5711 (75.6)	249 (71.8)	

### Association of cardiovascular health score at age 50 with incidence of dementia

**Fig 2:**
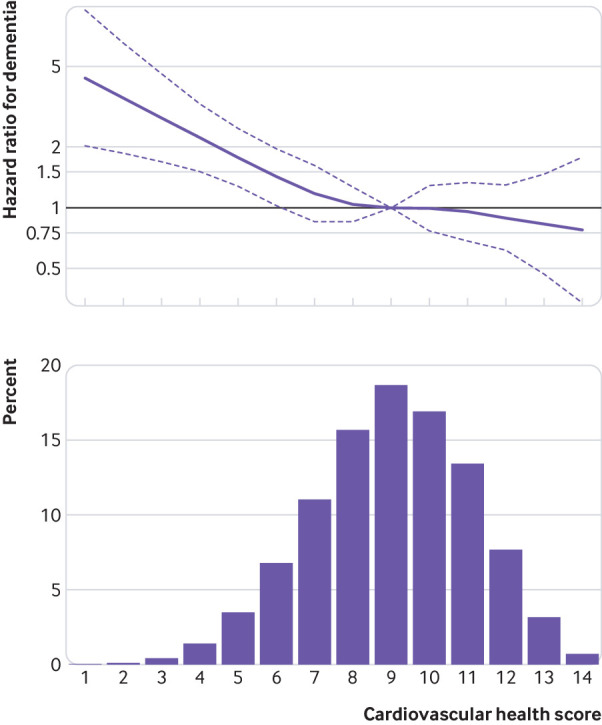
Association between continuous 14 point cardiovascular health score and incidence of dementia. Hazard ratio (95% CI) was estimated using inverse probability weighted Cox regression model with age as timescale and adjusted for sex, ethnicity, education, occupational position, and marital status, with a score of 9 (mean) as reference (hazard ratio=1)

As we found no evidence of effect modification by sex (P for interaction=0.42), we did analyses in men and women combined, with adjustment for sex. We verified the proportional hazard assumption by using Schoenfeld residuals and Kaplan-Meier curves (supplementary figure A). Figure 2 shows the association between the 14 point cardiovascular health score and risk of dementia to be linear (P for non-linearity=0.26). Further analyses using cardiovascular health score categorised in three groups showed the incidence rate of dementia to be lower in the intermediate and optimal cardiovascular health groups than in the poor cardiovascular health group (table 3). Compared with an incidence rate of dementia of 3.2 (95% confidence interval 2.5 to 4.0) per 1000 person years among the group with poor cardiovascular health (scores 0-6), the absolute rate differences per 1000 person years were −1.5 (95% confidence interval −2.3 to −0.7) for the group with intermediate (7-11) and −1.9 (−2.8 to −1.1) for the group with optimal (12-14) cardiovascular health. In an analysis adjusted for sociodemographic factors, compared with the poor cardiovascular health category, intermediate and optimal categories of cardiovascular health were associated with lower risk of dementia (hazard ratio 0.61 (0.47 to 0.80) and 0.57 (0.36 to 0.90), respectively; table 3). The hazard ratio for dementia for an increment of 1 point in the cardiovascular health score was 0.89 (0.85 to 0.95). Additional adjustment for cardiovascular disease as a time varying variable slightly attenuated this association (hazard ratio 0.91 (0.86 to 0.97) per 1 point increment in the cardiovascular health score).

**Table 3:** Cardiovascular health score and incidence of dementia (median follow-up 24.7 (interquartile range 19.1-29.3) years)

	No of cases/total No	Incidence rate (95% CI) per 1000 person years	Hazard ratio (95% CI) for dementia: model 1*	P value	Hazard ratio for dementia (95% CI): model 1+CVD†	P value
**Cardiovascular health score**						
Poor (0-6)	71/978	3.2 (2.5 to 4.0)	1 (reference)		1 (reference)	
Intermediate (7-11)	249/5997	1.8 (1.5 to 2.0)	0.61 (0.47 to 0.80)	<0.001	0.67 (0.51 to 0.88)	0.004
Optimal (12-14)	27/924	1.3 (0.8 to 1.8)	0.57 (0.36 to 0.90)	0.02	0.65 (0.41 to 1.03)	0.06
1 point increment in CVH score (range 0-14)	347/7899	1.9 (1.7 to 2.1)	0.89 (0.85 to 0.95)	<0.001	0.91 (0.86 to 0.97)	0.001
Each additional CVH metric at optimal level (range 0-7)	347/7899	1.9 (1.7 to 2.1)	0.88 (0.79 to 0.97)	0.01	0.90 (0.81 to 0.99)	0.04
**Cardiovascular health subscales**						
1 point increment in behavioural scale (range 0-8)	347/7899	1.9 (1.7 to 2.1)	0.87 (0.81 to 0.93)	<0.001	0.89 (0.82 to 0.95)	0.001
1-point increment in biological scale (range 0-6)	347/7899	1.9 (1.7 to 2.1)	0.91 (0.83 to 1.00)	0.06	0.94 (0.86 to 1.04)	0.24

CVD=cardiovascular disease; CVH=cardiovascular health.

* Hazard ratio estimated using inverse probability weighted Cox regression models with age as timescale and adjusted for sex, ethnicity, education, occupational position, and marital status (model 1).

† Model 1, additionally adjusted for time dependent cardiovascular disease.

In an analysis using the seven cardiovascular health metrics at optimal levels, and adjusted for sociodemographic factors, the hazard ratio for dementia for each additional metric at optimal level was 0.88 (0.79 to 0.97). Both the behavioural and biological subscale scores were associated with incidence of dementia (hazard ratio per 1 point increment 0.87 (0.81 to 0.93) and 0.91 (0.83 to 1.00), respectively), and we found no evidence of an interaction between the two (P=0.32). On further analysis in 4996 participants with relevant data available, results remained similar after additional adjustment for apolipoprotein E ε4 (supplementary table B).

### Role of cardiovascular health score at age 50 in transitions between healthy state, cardiovascular disease, and dementia

**Fig 3:**
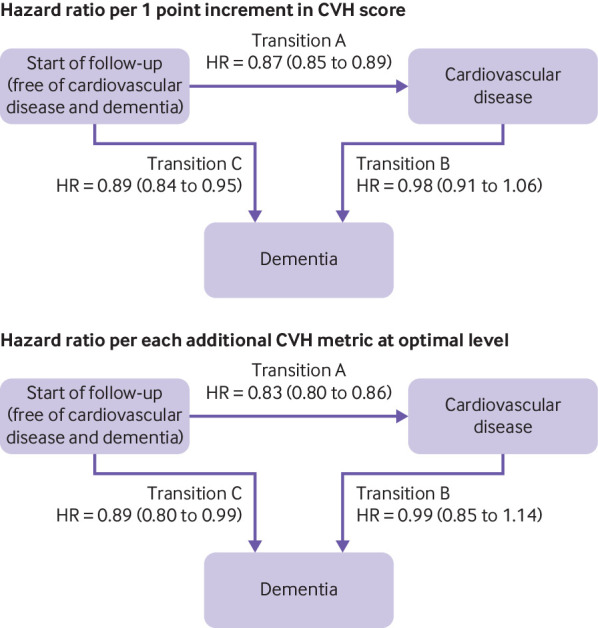
Multi-state models for role of cardiovascular health (CVH) score in transitions between start of follow-up, cardiovascular disease, and dementia. Top: hazard ratio (HR) per 1 point increment in CVH score. Bottom: HR per each additional CVH metric at optimal level (range 0-7). HRs and 95% CIs were estimated using inverse probability weighted multi-state models with age as timescale and adjusted for sex, ethnicity, education, occupational position, and marital status. No of cardiovascular disease (CVD; coronary heart disease or stroke) cases among healthy participants=1570/7899 (transition A); No of dementia cases among healthy participants=222/7899 (transition B); No of dementia cases among participants with CVD over follow-up=125/1570 (transition C)

Before use of multi-state modelling transitions between health states, we examined the association between cardiovascular disease and incidence of dementia. As expected, cardiovascular disease was associated with a higher risk of incident dementia (hazard ratio 1.96, 1.56 to 2.48) in a Cox regression with cardiovascular disease treated as a time dependent variable (data not tabulated). Figure 3 shows results from multi-state models in participants free of dementia and cardiovascular disease at age 50 (n=7899). A 1 point increment in the cardiovascular health score was associated with a reduced risk of incident cardiovascular disease over the follow-up (transition A: hazard ratio 0.87, 0.85 to 0.89). Among people free of cardiovascular disease before diagnosis of dementia, a 1 point increment in cardiovascular health score was associated with a lower risk of dementia (transition B: hazard ratio 0.89, 0.84 to 0.95), suggesting that the association between cardiovascular health and dementia is not fully explained by clinically manifest cardiovascular disease. In those with cardiovascular disease over the follow-up, cardiovascular health score at age 50 was not associated with risk of dementia following diagnosis of cardiovascular disease (transition C: hazard ratio 0.98, 0.91 to 1.06).

### Association of cardiovascular health at age 50 with brain volume 20 years later

Among 771 participants who had magnetic resonance imaging assessment, data on cardiovascular health at age 50 were available for 708 participants (569 (80.4%) men, mean age at assessment 69.9 (SD 5.1) years). One year higher age was associated with 0.33% (95% confidence interval 0.30% to 0.36%) lower whole brain volume, 0.25% (0.22% to 0.28%) lower grey matter volume, 0.08% (0.06% to 0.10%) lower white matter volume, 0.09 (0.07 to 0.10) points higher white matter hyperintensities on the Fazekas scale, and 0.11 (0.09 to 0.13) points greater hippocampal atrophy on the Scheltens score. Higher cardiovascular health score (per 1 point increment in the cardiovascular health score) was associated with 0.14% (0.06% to 0.22%) higher total brain and 0.12% (0.06% to 0.19%) higher grey matter volumes (table 4), corresponding to an age effect of 5.1 months on whole brain volume and 5.8 months on grey matter volume. Higher cardiovascular health score was associated with lower hippocampal atrophy, but the results did not reach statistical significance (P=0.07).

**Table 4:** Association between cardiovascular health (CVH) score at age 50 and structural brain volume 19.7 (SD 4.9) years later: Whitehall II substudy (n=708)

	No	Difference* (95% CI)	P value
**Total brain volume (% total intracranial volume)**			
CVH score:			
Poor (0-6)	58	0 (reference)	
Intermediate (7-11)	532	1.20 (0.59 to 1.80)	<0.001
Optimal (12-14)	118	1.30 (0.60 to 2.00)	<0.001
1 point increment in CVH score (range 0-14)	708	0.14 (0.06 to 0.22)	<0.001
Each additional CVH metric at optimal level (range 0-7)	708	0.23 (0.11 to 0.35)	<0.001
**Grey matter volume (% total intracranial volume)**			
CVH score:			
Poor (0-6)	58	0 (reference)	
Intermediate (7-11)	532	0.73 (0.23 to 1.23)	0.004
Optimal (12-14)	118	0.93 (0.35 to 1.52)	0.002
1 point increment in CVH score (range 0-14)	708	0.12 (0.06 to 0.19)	<0.001
Each additional CVH metric at optimal level (range 0-7)	708	0.20 (0.10 to 0.30)	<0.001
**White matter volume (% total intracranial volume)**			
CVH score:			
Poor (0-6)	58	0 (reference)	
Intermediate (7-11)	532	0.46 (0.04 to 0.89)	0.03
Optimal (12-14)	118	0.37 (−0.13 to 0.86)	0.15
1 point increment in CVH score (range 0-14)	708	0.02 (−0.04 to 0.08)	0.51
Each additional CVH metric at optimal level (range 0-7)	708	0.03 (−0.06 to 0.12)	0.50
**Total white matter hyperintensities (total Fazekas score, range 0-6)**			
CVH score:			
Poor (0-6)	58	0 (reference)	
Intermediate (7-11)	532	−0.12 (−0.46 to 0.23)	0.51
Optimal (12-14)	118	−0.19 (−0.60 to 0.21)	0.34
1 point increment in CVH score (range 0-14)	708	−0.02 (−0.07 to 0.02)	0.31
Each additional CVH metric at optimal level (range 0-7)	708	−0.04 (−0.11 to 0.03)	0.28
**Hippocampal atrophy (Scheltens score, range 0-8)**			
CVH score:			
Poor (0-6)	58	0 (reference)	
Intermediate (7-11)	532	−0.19 (−0.55 to 0.16)	0.29
Optimal (12-14)	118	−0.39 (−0.80 to 0.03)	0.07
1 point increment in CVH score (range 0-14)	708	−0.04 (−0.09 to 0.00)	0.07
Each additional CVH metric at optimal level (range 0-7)	708	−0.07 (−0.14 to 0.01)	0.08

* Linear regression models adjusted for age, sex, ethnicity, education, occupational position, marital status, and magnetic resonance imaging scanner.

## Discussion

This longitudinal study based on 7899 men and women has three key findings. Firstly, the American Heart Association’s cardiovascular health score assessed at age 50 was associated with subsequent risk of dementia over a 24.7 year median follow-up period, with lower risk observed for both intermediate and optimal scores compared with poor scores. The associations were evident for the continuous cardiovascular health score as well as the behavioural and biological scores, suggesting that both these subscales contribute to dementia risk. Secondly, the association between cardiovascular health at age 50 and subsequent dementia was also observed among people without coronary heart disease or stroke during the follow-up period. Thus, cardiovascular disease events do not fully account for the association between cardiovascular health and dementia. Thirdly, findings were corroborated in results using magnetic resonance imaging data for a subcohort. Higher cardiovascular health score at age 50 was associated with higher whole brain and grey matter volumes 20 years later.

### Strengths and limitations of study

We assessed cardiovascular health close to age 50 (mean age 50.4 (SD 2.3) years, age range 45-55). Much of the previous evidence on “midlife” risk factors has used a wide age range, sometimes as large as 20 years, from a baseline assessment to draw conclusions on the importance of midlife factors. This is particularly problematic for research on dementia, as risk factor levels change during the long preclinical period of dementia,^1^ which can lead to findings that are affected by reverse causation.^12^ ^13^ ^22^ Key strengths of this study include use of a study design that allowed us to assess cardiovascular health metrics at age 50, a long follow-up for incidence of dementia, the use of multiple statistical methods to control for bias and confounding,^21^ and inclusion of data from the Whitehall II magnetic resonance imaging substudy.

A key limitation, as in any observational study, is the self reported measure of behavioural factors and drug use. The Whitehall II study is an occupational cohort at recruitment, and participants are healthier than the general population in terms of risk factors levels and incidence of disease. However, this does not necessarily affect risk factor-disease associations.^23^ For example, the association between cardiovascular risk factors and incidence of cardiovascular disease in the Whitehall II study is similar to that in general population studies.^24^

Use of linkage to electronic health records for ascertainment of dementia may not be the “gold standard” method but has the advantage of allowing analysis on everyone recruited to the study rather than only those who continue to participate over the course of the study and are available for an in-person clinical ascertainment of dementia. In the UK, HES records on dementia have been shown to have high specificity but modest sensitivity (78%) due to missing of milder cases of dementia,^15^ as also reported in the Mayo Clinic Study of Ageing and the Adult Changes study.^25^ We additionally used the UK mental health database to improve the sensitivity of dementia diagnosis.^26^

### Comparison with previous studies

Few previous studies have examined the association between cardiovascular health and cognitive outcomes, and most were based on older adults who were followed for cognitive outcomes for less than 10 years. These studies reported better cardiovascular health to be associated with lower risk of cognitive impairment,^27^ cognitive decline,^8^ ^9^ and dementia.^8^ ^10^ In the Atherosclerosis Risk in Community (ARIC) cohort, higher cardiovascular health scores assessed at mean age 54 (SD 6) years were associated with slower cognitive decline over a 20 year follow-up period.^28^ In participants from the Framingham Heart Study Offspring cohort, the association with risk of dementia was observed when risk factors were assessed at a mean age of 62 (SD 6) years but not when assessed at a mean age of 69 (6) years.^10^ In a study on people with a mean age of 67 (8) years at cardiovascular health assessment, no association was found with incidence of dementia over a seven year mean follow-up.^11^ One explanation for this inconsistency is that studies that assess risk factors at older ages are more likely to be affected by reverse causation bias than are those with this assessment in midlife, a possible explanation for null findings in recent studies on cardiovascular health and dementia.^10^ ^11^ Evidence shows that the associations of body mass index, blood pressure, and cholesterol with dementia are attenuated or even reversed at older ages.^11^ ^12^ ^13^ ^29^ ^30^ ^31^

Few studies, mostly cross sectional, have examined the association between cardiovascular health and brain imaging.^10^ ^26^ ^32^ ^33^ Cardiovascular health has been shown to be associated with whole brain volume,^10^ ^32^ although findings on specific markers are less consistent.^10^ ^26^ ^32^ ^33^ White matter hyperintensities, particularly in the deep white matter, are usually interpreted as “microvascular changes” that are associated with vascular risk in general.^34^ We found no association with cardiovascular health, which is consistent with some but not all previous studies.^10^ ^32^ ^33^ ^35^ The large heterogeneity in population settings (mean age ranged from young adulthood to older age), as well as the type of brain imaging analysis, might explain inconsistent findings. Further investigations are thus needed to confirm specific brain correlates of cardiovascular health.

### Meaning of study

The AHA’s recently defined concept of “ideal cardiovascular health” by presence of all seven metrics at an ideal level is useful for the primordial prevention of cardiovascular disease, as shown in several studies.^5^ In our data, each additional cardiovascular health metric at optimal level was more strongly associated with a lower risk of cardiovascular diseases than was a 1 point increment in the more refined 14 point cardiovascular health score (fig 3, transition A). As a similar tool does not exist for dementia, we used the cardiovascular health metrics at an optimal level and the 14 point cardiovascular health score.

Our results showed reductions in the risk of dementia across the continuum of the 14 point cardiovascular health score, highlighting the importance of clustering of cardiovascular risk factors in midlife for risk of dementia at older ages. Each 1 point increment in the 14 point cardiovascular health score was associated with a similar reduction in the risk of dementia to each additional cardiovascular health metric at an optimal level (seven metrics). Use of a finely graded 14 point scale, which also considers each metric at an intermediate level, suggests that even small improvements in cardiovascular health metrics, without necessarily reaching the optimal level for each metric, are likely to be beneficial in reducing the risk of dementia. From a public health perspective, moving from a poor to an intermediate level in cardiovascular health metrics is perhaps more achievable and sustainable in the long term than moving from a poor to an optimal level. In addition, both the behavioural and biological subscales of the cardiovascular health score were associated with incidence of dementia, and the absence of an interaction between the two subscales highlights the importance of the behavioural score at all levels of the biological subscale, including among people without biological risk factors at age 50. Dementia has a multifactorial aetiology, and accordingly multidomain interventions targeting several risk factors simultaneously have been recommended for prevention.^36^ Although the cardiovascular health metrics were not originally developed for the prevention of dementia, results from this observational study suggest that their assessment in midlife could be used for identification of groups at increased risk of dementia and that interventions targeting cardiovascular risk factors in midlife might additionally decrease the risk of dementia.^3^ ^7^

Use of multi-state models in this study showed a robust association between cardiovascular health and incidence of dementia in participants free of major cardiovascular disease over the follow-up. Possible mechanisms by which cardiovascular health is associated with risk of dementia therefore include mechanisms beyond occlusion of major arteries, such as cerebral small vessel lesions, hypoxia, microinfarctions, inflammation, oxidative stress, and advanced glycation end products.^37^ The precise pathway through which vascular and degenerative processes determine risk of Alzheimer’s disease and dementia is likely to be complex. Our results showing cardiovascular health assessed at age 50 to be associated with dementia and brain volume 20 years later are in agreement with previous studies based on shorter follow-up.^10^ ^33^ These results highlight the importance of the cardiovascular health score in promoting brain health at older ages.

### Conclusion

Prevention is an important element in tackling the challenge posed by the expected tripling of dementia cases by 2050. Our findings suggest that the Life’s Simple 7, which comprises the cardiovascular health score, at age 50 may shape the risk of dementia in a synergistic manner. Cardiovascular risk factors are modifiable, making them strategically important prevention targets. This study supports public health policies to improve cardiovascular health as early as age 50 to promote cognitive health.

**What is already known on this topic**

Dementia is a progressive multifactorial disease involving pathophysiological changes over a long preclinical period
The Life Simple 7 cardiovascular health score has been put forward as a potential tool for prevention of dementia, although evidence from observational studies remains inconsistent
An important source of inconsistency in the findings stems from the fact that most studies assess risk factors in late life and examine the onset of dementia over short follow-up periods
Findings are thus likely to be attributable to reverse causation due to changes in risk factor levels in the preclinical phase of dementia

**What this study adds**

Adherence at age 50 to the Life Simple 7 cardiovascular health recommendations, designed for the prevention of cardiovascular disease, might also decrease risk of dementia at older age
This association was also observed in people who remained free of cardiovascular disease over the follow-up
Reductions in risk of dementia were evident across the continuum of the cardiovascular health score, suggesting that even small improvements in cardiovascular risk factors are likely to be beneficial for cognitive health

## Web extra

Extra material supplied by authors

## Notes

Contributors: ASM and SS developed the hypothesis and study design. SS, AF, and AD did statistical analysis. SS wrote the first and successive drafts of the manuscript. All authors contributed to study concept and design, analysis and interpretation of data, and drafting or critical revision of the manuscript for important intellectual content or, in addition, data acquisition. ASM, MK, and KPE obtained funding. The corresponding author attests that all listed authors meet authorship criteria and that no others meeting the criteria have been omitted. SS is the guarantor.

Ethical approval: Written informed consent from participants and research ethics approvals were renewed at each contact; the most recent approval was from the University College London Hospital Committee on the Ethics of Human Research, reference number 85/0938.

Data Sharing: Whitehall II data, protocols, and other metadata are available to the scientific community. Please refer to the Whitehall II data sharing policy at https://www.ucl.ac.uk/epidemiology-health-care/research/epidemiology-and-public-health/research/whitehall-ii/data-sharing.

Transparency declaration: The lead author (the manuscript’s guarantor) affirms that this manuscript is an honest, accurate, and transparent account of the study being reported; that no important aspects of the study have been omitted; and that any discrepancies from the study as planned (and, if relevant, registered) have been explained.

## References

[1] Jack CR Jr Knopman DS Jagust WJ Tracking pathophysiological processes in Alzheimer’s disease: an updated hypothetical model of dynamic biomarkers. Lancet Neurol 2013;12:207-16. 10.1016/S1474-4422(12)70291-0 23332364 PMC3622225

[2] Larson EB Yaffe K Langa KM New insights into the dementia epidemic. N Engl J Med 2013;369:2275-7. 10.1056/NEJMp1311405 24283198 PMC4130738

[3] Winblad B Amouyel P Andrieu S Defeating Alzheimer’s disease and other dementias: a priority for European science and society. Lancet Neurol 2016;15:455-532. 10.1016/S1474-4422(16)00062-4 26987701

[4] Lloyd-Jones DM Hong Y Labarthe D American Heart Association Strategic Planning Task Force and Statistics Committee Defining and setting national goals for cardiovascular health promotion and disease reduction: the American Heart Association’s strategic Impact Goal through 2020 and beyond. Circulation 2010;121:586-613. 10.1161/CIRCULATIONAHA.109.192703 20089546

[5] Fang N Jiang M Fan Y Ideal cardiovascular health metrics and risk of cardiovascular disease or mortality: A meta-analysis. Int J Cardiol 2016;214:279-83. 10.1016/j.ijcard.2016.03.210 27085116

[6] Joseph JJ Echouffo-Tcheugui JB Carnethon MR The association of ideal cardiovascular health with incident type 2 diabetes mellitus: the Multi-Ethnic Study of Atherosclerosis. Diabetologia 2016;59:1893-903. 10.1007/s00125-016-4003-7 27272340 PMC4970884

[7] Gorelick PB Furie KL Iadecola C American Heart Association/American Stroke Association Defining Optimal Brain Health in Adults: A Presidential Advisory From the American Heart Association/American Stroke Association. Stroke 2017;48:e284-303. 10.1161/STR.0000000000000148 28883125 PMC5654545

[8] Samieri C Perier MC Gaye B Association of Cardiovascular Health Level in Older Age With Cognitive Decline and Incident Dementia. JAMA 2018;320:657-64. 10.1001/jama.2018.11499 30140876 PMC6142948

[9] Gardener H Wright CB Dong C Ideal Cardiovascular Health and Cognitive Aging in the Northern Manhattan Study. J Am Heart Assoc 2016;5:e002731. 10.1161/JAHA.115.002731 26984255 PMC4943249

[10] Pase MP Beiser A Enserro D Association of Ideal Cardiovascular Health With Vascular Brain Injury and Incident Dementia. Stroke 2016;47:1201-6. 10.1161/STROKEAHA.115.012608 27073239 PMC5006676

[11] Hessler JB Ander KH Brönner M Predicting dementia in primary care patients with a cardiovascular health metric: a prospective population-based study. BMC Neurol 2016;16:116. 10.1186/s12883-016-0646-8 27459854 PMC4962452

[12] Kivimäki M Luukkonen R Batty GD Body mass index and risk of dementia: Analysis of individual-level data from 1.3 million individuals. Alzheimers Dement 2018;14:601-9. 10.1016/j.jalz.2017.09.016 29169013 PMC5948099

[13] Abell JG Kivimäki M Dugravot A Association between systolic blood pressure and dementia in the Whitehall II cohort study: role of age, duration, and threshold used to define hypertension. Eur Heart J 2018;39:3119-25. 10.1093/eurheartj/ehy288 29901708 PMC6122131

[14] Marmot MG Smith GD Stansfeld S Health inequalities among British civil servants: the Whitehall II study. Lancet 1991;337:1387-93. 10.1016/0140-6736(91)93068-K 1674771

[15] Sommerlad A Perera G Singh-Manoux A Lewis G Stewart R Livingston G Accuracy of general hospital dementia diagnoses in England: Sensitivity, specificity, and predictors of diagnostic accuracy 2008-2016. Alzheimers Dement 2018;14:933-43. 10.1016/j.jalz.2018.02.012 29703698 PMC6057268

[16] Filippini N Zsoldos E Haapakoski R Study protocol: The Whitehall II imaging sub-study. BMC Psychiatry 2014;14:159. 10.1186/1471-244X-14-159 24885374 PMC4048583

[17] Fazekas F Chawluk JB Alavi A Hurtig HI Zimmerman RA MR signal abnormalities at 1.5 T in Alzheimer’s dementia and normal aging. AJR Am J Roentgenol 1987;149:351-6. 10.2214/ajr.149.2.351 3496763

[18] Scheltens P Leys D Barkhof F Atrophy of medial temporal lobes on MRI in “probable” Alzheimer’s disease and normal ageing: diagnostic value and neuropsychological correlates. J Neurol Neurosurg Psychiatry 1992;55:967-72. 10.1136/jnnp.55.10.967 1431963 PMC1015202

[19] Harrell FE Jr Regression Modeling Strategies: With Applications to Linear Models, Logistic Regression, and Survival Analysis. Springer, 2001 10.1007/978-1-4757-3462-1.

[20] Orsini N Greenland S A procedure to tabulate and plot results after flexible modeling of a quantitative covariate. Stata J 2011;11:1-29 10.1177/1536867X1101100101.

[21] Weuve J Tchetgen Tchetgen EJ Glymour MM Accounting for bias due to selective attrition: the example of smoking and cognitive decline. Epidemiology 2012;23:119-28. 10.1097/EDE.0b013e318230e861 21989136 PMC3237815

[22] Sabia S Dugravot A Dartigues JF Physical activity, cognitive decline, and risk of dementia: 28 year follow-up of Whitehall II cohort study. BMJ 2017;357:j2709. 10.1136/bmj.j2709 28642251 PMC5480222

[23] Rothman KJ Gallacher JE Hatch EE Why representativeness should be avoided. Int J Epidemiol 2013;42:1012-4. 10.1093/ije/dys223 24062287 PMC3888189

[24] Batty GD Shipley M Tabák A Generalizability of occupational cohort study findings. Epidemiology 2014;25:932-3. 10.1097/EDE.0000000000000184 25265141

[25] Knopman DS Petersen RC Rocca WA Larson EB Ganguli M Passive case-finding for Alzheimer’s disease and dementia in two U.S. communities. Alzheimers Dement 2011;7:53-60. 10.1016/j.jalz.2010.11.001 21255743 PMC3137259

[26] Wilkinson T Ly A Schnier C UK Biobank Neurodegenerative Outcomes Group and Dementias Platform UK Identifying dementia cases with routinely collected health data: A systematic review. Alzheimers Dement 2018;14:1038-51. 10.1016/j.jalz.2018.02.016 29621480 PMC6105076

[27] Thacker EL Gillett SR Wadley VG The American Heart Association Life’s Simple 7 and incident cognitive impairment: The REasons for Geographic And Racial Differences in Stroke (REGARDS) study. J Am Heart Assoc 2014;3:e000635. 10.1161/JAHA.113.000635 24919926 PMC4309046

[28] González HM Tarraf W Harrison K Midlife cardiovascular health and 20-year cognitive decline: Atherosclerosis Risk in Communities Study results. Alzheimers Dement 2018;14:579-89. 10.1016/j.jalz.2017.11.002 29268079 PMC5938099

[29] Fitzpatrick AL Kuller LH Lopez OL Midlife and late-life obesity and the risk of dementia: cardiovascular health study. Arch Neurol 2009;66:336-42. 10.1001/archneurol.2008.582 19273752 PMC3513375

[30] Singh-Manoux A Dugravot A Shipley M Obesity trajectories and risk of dementia: 28 years of follow-up in the Whitehall II Study. Alzheimers Dement 2018;14:178-86. 10.1016/j.jalz.2017.06.2637 28943197 PMC5805839

[31] Tzourio C Laurent S Debette S Is hypertension associated with an accelerated aging of the brain? Hypertension 2014;63:894-903. 10.1161/HYPERTENSIONAHA.113.00147 24566080

[32] Bancks MP Allen NB Dubey P Cardiovascular health in young adulthood and structural brain MRI in midlife: The CARDIA study. Neurology 2017;89:680-6. 10.1212/WNL.0000000000004222 28724586 PMC5562970

[33] Gardener H Caunca M Dong C Ideal Cardiovascular Health and Biomarkers of Subclinical Brain Aging: The Northern Manhattan Study. J Am Heart Assoc 2018;7:e009544. 10.1161/JAHA.118.009544 30369305 PMC6201403

[34] Erten-Lyons D Woltjer R Kaye J Neuropathologic basis of white matter hyperintensity accumulation with advanced age. Neurology 2013;81:977-83. 10.1212/WNL.0b013e3182a43e45 23935177 PMC3888199

[35] Williamson W Lewandowski AJ Forkert ND Association of Cardiovascular Risk Factors With MRI Indices of Cerebrovascular Structure and Function and White Matter Hyperintensities in Young Adults. JAMA 2018;320:665-73. 10.1001/jama.2018.11498 30140877 PMC6142949

[36] Kivipelto M Mangialasche F Ngandu T Lifestyle interventions to prevent cognitive impairment, dementia and Alzheimer disease. Nat Rev Neurol 2018;14:653-66. 10.1038/s41582-018-0070-3 30291317

[37] Gorelick PB Scuteri A Black SE American Heart Association Stroke Council, Council on Epidemiology and Prevention, Council on Cardiovascular Nursing, Council on Cardiovascular Radiology and Intervention, and Council on Cardiovascular Surgery and Anesthesia Vascular contributions to cognitive impairment and dementia: a statement for healthcare professionals from the american heart association/american stroke association. Stroke 2011;42:2672-713. 10.1161/STR.0b013e3182299496 21778438 PMC3778669

