# Supplementary material for: Association of ideal cardiovascular health at age 50 with incidence of dementia: 25 year follow-up of Whitehall II cohort study
Source: BMJ. 2019 Jul 31;366:l4414. doi: 10.1136/bmj.l4414 (PMC6664261; doi:10.1136/bmj.l4414)
Supplement: Supplementary file 1 — Supplementary materials [file sabs049100.ww1.pdf]

SUPPLEMENTARY MATERIALS

Figure A. Probability of survival free of dementia by CVH score at age 50

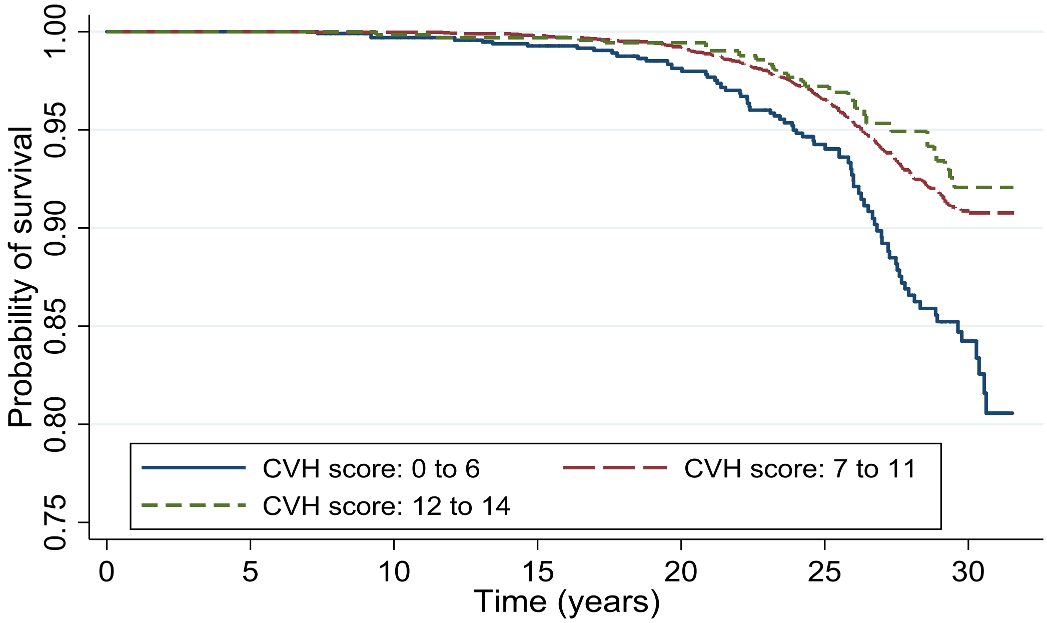

Years of follow-up	0-4.9	5-9.9	10-14.9	15-19.9	20-24.9	25-29.9	30-31.6
N dementia cases	0	4	15	36	127	160	5

Abbreviations: CVH, cardiovascular health.

Table A. Metrics distribution at age 50 (%)

	All N=7899	CVH score Poor (0-6) N=978	Intermediate (7-11) N=5997	Optimal (12-14) N=924
**Smoking**				
Poor	1239 (15.7)	490 (50.1)	747 (12.5)	2 (0.2)
Intermediate	313 (4.0)	71 (7.3)	230 (3.8)	12 (1.3)
Optimal	6347 (80.3)	417 (42.6)	5020 (83.7)	910 (98.5)
**Diet**				
Poor	2789 (35.3)	674 (68.9)	2068 (34.5)	47 (5.1)
Intermediate	3748 (47.5)	282 (28.8)	3081 (51.4)	382 (41.7)
Optimal	1362 (17.2)	22 (2.3)	848 (14.1)	492 (53.2)
**Physical activity**				
Poor	1310 (16.6)	479 (49.0)	820 (13.7)	11 (1.2)
Intermediate	2625 (33.2)	331 (33.8)	2123 (35.4)	171 (18.5)
Optimal	3964 (50.2)	168 (17.2)	3054 (50.9)	742 (80.3)
**BMI**				
Poor	849 (10.7)	352 (36.0)	495 (8.3)	2 (0.2)
Intermediate	3024 (38.3)	459 (46.9)	2464 (41.1)	101 (10.9)
Optimal	4026 (51.0)	167 (17.1)	3038 (50.7)	821 (88.9)
**Glucose**				
Poor	112 (1.4)	60 (6.1)	52 (0.9)	0 (0.0)
Intermediate	855 (10.8)	222 (22.7)	607 (10.1)	26 (2.8)
Optimal	6932 (87.8)	696 (70.2)	5338 (89.0)	898 (97.2)
**Cholesterol**				
Poor	3523 (44.6)	737 (75.4)	2742 (45.7)	44 (4.8)
Intermediate	2926 (37.0)	220 (22.5)	2339 (39.0)	367 (39.7)
Optimal	1450 (18.4)	21 (2.2)	916 (15.3)	513 (55.5)
**Blood pressure**				
Poor	1468 (18.6)	451 (46.1)	1010 (16.8)	7 (0.8)
Intermediate	3399 (43.0)	416 (42.5)	2797 (46.6)	186 (20.1)
Optimal	3032 (38.4)	111 (11.4)	2190 (36.5)	731 (79.1)

Abbreviations: BMI, body mass index; CVH, cardiovascular health.

Table B. CVH score and incidence of dementia, additional adjustment for APOE ε4 (N cases/N total = 176/4996)

	**HR for dementia (95%CI) Model 1***	**P**	**HR for dementia (95%CI) Model 1 + APOE ε4**	**P**
**CVH score**				
1-point increment in the CVH score (range, 0-14)	0.89 (0.82 to 0.96)	0.005	0.89 (0.82 to 0.97)	0.007
Each additional CVH metric at optimal level (range, 0-7)	0.86 (0.75 to 1.00)	0.05	0.87 (0.75 to 1.01)	0.08

Abbreviations: APOE, apolipoprotein E; CI, confidence interval; CVH, cardiovascular health; HR, hazard ratio.

*HR estimated among the 4996 participants with data on APOE, using inverse probability weighted Cox regression models with age as time-scale and adjusted for sex, ethnicity, education, occupational position, and marital status.
